# Cytochromes b5 Occurrence in Viruses Belonging to the Order Megavirales

**DOI:** 10.21203/rs.3.rs-5246363/v1

**Published:** 2024-10-25

**Authors:** David C. Lamb, Jared V. Goldstone, Djamal Brahim Belhaouari, Julien Andréani, Ayesha Farooqi, Michael J. Allen, Steven L. Kelly, Bernard La Scola, John J. Stegeman

**Affiliations:** 1 Faculty of Medicine, Health and Life Sciences, Swansea University, Swansea, Wales SA2 8PP, UK; 2 Biology Department, Woods Hole Oceanographic Institution, Woods Hole, Massachusetts 02543, USA; 3 Department of Veterinary Pathobiology, School of Veterinary Medicine & Biomedical Sciences, Texas A&M University, College Station, Texas, USA; 4 Aix Marseille Univ, MEPHI, Marseille, France; 5 IHU-Méditerranée infection, Marseille, France; 6 Department of Biosciences, College of Life and Environmental Sciences, University of Exeter, Stocker Road EX4 4QD, UK.

**Keywords:** Cytochrome b5, cytochrome P450, giant virus, evolution, amoeba, redox partner

## Abstract

Cytochrome b5 is a small electron transport protein that is found in animals, plants, fungi and photosynthetic proteobacteria where it plays key metabolic roles in energy production, lipid and sterol biosynthesis and cytochrome P450 biochemistry. Previously it was shown that a gene encoding a soluble and functional cytochrome b5 protein was encoded in the large double stranded DNA virus OtV2 that infects the unicellular marine green alga *Ostreococcus tauri*, the smallest free-living eukaryote described to-date. This single gene represented a unique finding in the virosphere. We now report that genes for soluble and membrane-bound cytochromes b5 also occur in giant viruses in the proposed order Megavirales, particularly the AT-rich *Mimiviridae* and *Tupanviruses*. Conversely, other members of the *Megavirales* taxa such as the GC-rich *Pandoraviridae* have not been found to encode cytochrome b5 as yet. Megaviruses encoding cytochrome b5 have been isolated from the deep ocean, from freshwater and terrestrial sources, as well as from human patients. Giant virus cytochrome b5 proteins share high sequence identity with one another (45–95% depending on group) but no more than 25% identity with the cytochrome b5 gene product we identified in *Acanthamoeba castellanii*, an amoeba host for many giant viruses. Thus, the origin of the unique cytochrome b5 genes in giant viruses remains unknown. Examination of viral cytochrome b5 primary amino acid sequences revealed that some have either a N- or C-terminal transmembrane anchor, whilst others lack a membrane anchor and are thus predicted to be soluble proteins. This cytochrome b5 topography suggests adapted biochemical functions in those viruses. Our findings raise questions regarding the evolution and diversity of cytochrome b5 proteins in nature, adding to questions about the origin of viral haemoproteins in general.

## 1. Introduction

Giant viruses, discovered just over 20 years ago, are enigmatic members of the global microbiome ^1^. Traditionally, viruses were defined as biological entities on the edge of life, lacking cellular structure and metabolism, and only reproducing in a host cell. That view of viruses changed in 2003 with the discovery of a giant virus, *Acanthamoeba polyphaga Mimivirus* (APMV or Mimivirus) ^2^. The Mimivirus genome epitomized an emerging and diverse group of viruses, the nucleocytoplasmic large DNA viruses (NCLDV). Giant viruses, many larger than 0.2 μm and with genomes greater than 300 kbp, continue to be discovered in - or captured with - amoebae ^3^, and have been identified in a wide range of hosts, including algae ^4^. They are increasingly found in metagenomic datasets as well ^5,6^. With the growing number of giant viruses, a new order was proposed, the Megavirales ^1,7^, and more recently, a new phylum, the *Nucleocytoviricota* ^8^. Especially prominent to-date are members of the *Mimiviridae, w*ith AT-rich DNA genomes, and the *Pandoraviridae*, which have GC-rich DNA genomes. Multiple species and clades occur in both families. They have been isolated from marine, freshwater, and/or terrestrial sources in different parts of the world ^9^.

The first giant virus discovered, Mimivirus, encodes more than 900 proteins, but fewer than 300 have predicted functions ^10^. Many Mimivirus sequences encode proteins not found in any other virus, including protein translation enzymes (thought to be a signature of cellular organisms), DNA repair pathway components and putative homologues of enzymes involved in eukaryotic metabolism ^10^. Since publication of the Mimivirus genome, numerous other metabolic genes thought only to be found in cellular lifeforms have been found in diverse GV genomes from different environments. Using bioinformatic approaches, we and others have identified GV genes and proteins and their orthologues that are involved in known cellular primary metabolic pathways *e.g.* glycolysis, gluconeogenesis, TCA cycle, photosynthesis and lipid β-oxidation ^11–13^. It is already accepted that viruses can hijack host metabolic networks, but the GVs auxiliary metabolic genes represent another form of host metabolism manipulation, by expanding, rather than merely enhancing, host cells’ catalytic capabilities especially in harsh variable environments. However, the functionality of these genes remains unclear, and how they integrate into established host metabolic systems is completely unknown ^14^.

**Figure 1.**
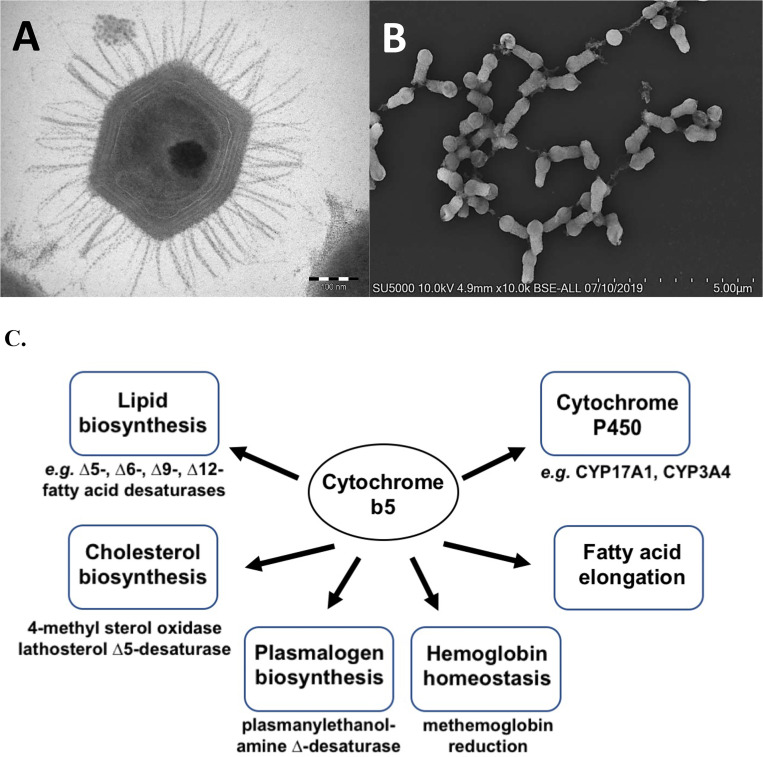
A. Electron microscopy image of Mimivirus particle. B. Scanning electron microscopy image of Tupanvirus viral particles. C. Cytochrome b5 redox partners and pathways. Arrows indicate electron flow. HMOX, heme oxygenase (2 genes); SQLE, squalene monooxygenase; CYP, cytochromes P450 (51 microsomal forms); CYB5, cytochrome b5 (2 genes); MSMO, methylsterol monooxygenase; SC5DL, sterol-C5-desaturase; SCD, stearoyl-CoA desaturase (2 genes); ELOVL, fatty acid elongase (7 genes).

Heme proteins are among the most versatile of metalloproteins found in nature and play essential roles in diverse and distinct biological functions from gas exchange to redox reductions ^15^. Cytochrome b5, originally detected in the larvae of the silkworm *Platysumia cecropia* by Sanborn and Williams in 1950 ^16^, is a ubiquitous electron transport protein found in animals, plants, fungi and purple photosynthetic bacteria ^17^. Soluble forms of cytochrome b5 function in photosynthetic energy production in bacteria and the reduction of hemoglobin in animals ^17,18^. In contrast, membrane-bound forms of cytochrome b5 function in the mitochondria and the endoplasmic reticulum (ER) of eukaryotes. ER-associated cytochrome b5 roles are shown in Figure 1 and include roles in fatty acid biosynthesis driving fatty acid desaturase enzymes (*Δ*9-, *Δ*6-, *Δ*5- and *Δ*12-desaturases), in cholesterol biosynthesis, and in the reduction of cytochromes P450 enzymes where cytochrome b5 has been shown to alter substrate metabolism by different P450s ^17^. No function has been attributed to cytochrome b5 in the mitochondria ^19^.

We previously cloned, expressed, and characterized a gene encoding a putative cytochrome b5 protein from the large double-stranded DNA virus *Ostreococcus tauri* virus OtV-2 ^20^. This soluble viral cytochrome b5 protein had near identical spectral properties to purified membrane-bound, recombinant human cytochrome b5 ^20^. The crystal structure of the *O. tauri* OtV-2 cytochrome b5 revealed a single domain, comprising four β sheets, four α helices and a heme moiety, which is similar to that found in larger eukaryotic cytochrome b5 proteins ^20^. The *O. tauri* OtV-2 cytochrome b5 also was shown to reduce a eukaryotic cytochrome P450 (*Candida albicans* CYP51) through the transfer of reducing equivalents, suggesting this viral cytochrome b5 can act as a *bona fide* cytochrome b5 in P450 reactions, as well as potentially be involved in modulating lipid and sterol biosynthetic enzyme activity. This added credence to our current hypothesis that novel viral heme proteins, including viral cytochrome P450 enzymes ^21^, can modulate either viral or host sterol or lipid content to maximize energy production and facilitate viral replication. This possibility has particular relevance to viruses that have lipid envelopes and/or rely on controlled passage through the membrane for virion release.

The current work describes the discovery of multiple cytochrome b5 enzymes in numerous giant virus genomes from metagenome datasets that are currently publicly available. Identification of enzyme systems that seemingly cannot function in the inert virion particle yet are identifiable across a wide range of viral species and ecotypes, points to key aspects of NCLDV biology that remain unexplored.

## 2. Materials and Methods

### 2.1 Viral Cytochrome b5 Sequence Datasets.

The cytochrome b5 domain is readily identifiable bioinformatically. Candidate viral cytochrome b5 sequences were retrieved from the NCBI sequence databases (years ??- JED?). Inferred protein sequences were aligned to the PfAM b5-domain hidden Markov model (PF00173) using ClustalOmega ^22^, and scored with T-Coffee ^23^. Alignments were visualized in BioEdit ^24^. Virus names are those in the original reports or described in Genbank. Pairwise amino acid identities reported in the text are based on the length of the shortest protein in the alignment. Identity values used in the tables are based on multiple sequence alignments and are usually not more than 1% different from stated values.

### 2.2 Phylogenetic Analyses of Viral Cytochrome b5.

Phylogenetic trees of cytochrome b5 were inferred using maximum likelihood (ML) approaches (RAxML v8.2.6) with the PROTCATLG model of amino acid substitution as selected by ModelTest.^25^ Twenty replicate ML searches were conducted, and the best-scoring tree was subjected to rapid bootstrapping in RAxML to assess bifurcation support using the autoMRE bootstopping criteria. Virus phylogenies were constructed using RAxML-NG from the conserved RNA Pol2 (RNAP2) gene using the PROTCATHIVB model of amino acid substitution ^21^. Rapid bootstrapping with the autoMRE bootstopping criteria produced 700 bootstrap replicates.

### 2.3 Modelling and structural analysis.

Homology modelling of viral b5 proteins was performed using Modeller (v9.16) ^26^. 100 independent models were produced based on rat, housefly, and *Ostreococcus* virus cytochrome b5 structures (PDB codes 4B8N,1EUE, 3MUS, 2IBJ). The model with the highest DOPE-HR (high resolution discrete optimized potential energy) score was selected and visualized in Pymol. Further structural analysis was performed using Thesesus ^27^. Subcellular colocalization of viral cytochrome b5 proteins was predicted using the DeepLoc 1.0 and DeepLoc 2.0 algorithms ^28^.

### 2.4 Mimivirus cytochrome b5 transcriptomic analysis and protein characterisation.

The Mimivirus cytochrome b5 gene (MIMI_L628) was synthesised by Eurofins MWG Operon and gene integrity confirmed by DNA sequencing. A unique *Nde*I restriction site was generated prior to the ATG start codon and a *Hin*dIII site following the TGA stop codon to facilitate cloning into the *Escherichia coli* expression plasmid, pET17b. Additionally, a polyhistidine tag, to allow purification of the expressed protein by Ni^2+^-NTA affinity chromatography, was engineered into the C-terminus of the protein. MIMI L628 was expressed and purified using similar conditions described for *Ostreococcus* virus cytochrome b5 [18]. Cytochrome b5 content was quantified from the reduced versus oxidized difference spectrum of purified protein using a differential absorption coefficient Δ_424 – 409_ of 185 mM^−1^ cm^−1 29^.

## 3. Results

### 3.1 Overall distribution of cytochrome b5 in the virosphere

**Figure 2.**
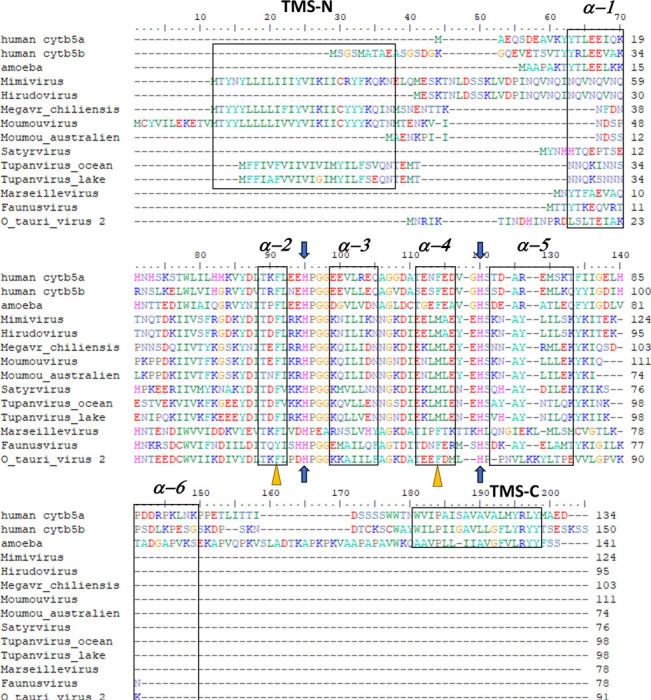
Sequence alignment of viral cytochromes b5. Alignment of amino acid sequences of human cytochrome b5a (endoplasmic reticulum) and human cytochrome b5b (mitochondrial) with amoeba (*A. castellanii*) cytb5, and various Megavirales cytb5, as well as previously reported *Ostreococcus tauri virus 2* cytochrome b5. The schematic arrangement of secondary structures of the proteins obtained from cytochrome b5 structures (PDB ID: 4HIL, 1AW3) is shown above the alignment. N- and C- terminal transmembrane regions of the proteins are indicated respectively as TMS-N and TMS-C. Blue arrows denote the heme-bound histidine residues, and the orange triangles show the important conserved heme-adjacent F40 and F63 (or M, see text). See also Figure 8. Generally conserved negatively charged residues at alignment positions 94–95, 99–100, 109, and 116 have been previously shown to interact with cytochrome P450 enzymes.

Among the viruses, we identified numerous cytochrome b5 genes encoded in the genomes of specific taxa of the *Megavirales* including *Ostreococcus* viruses, which are atypical giant viruses, based on the absence of RNAPol2. Genes were assigned as encoding cytochrome b5 based on the conservation of sequence and secondary structure identity with known eukaryotic cytochromes b5. Key factors in sequence assignment include the retention of secondary structure elements occurring in the order β1-α1-β4-β3-α2-α3-β5-α4-α5-β2-α6, the absolute conservation of two histidine residues residing in the loops of between helices α2 and α3 and between helices α4 and α5, and the overall domain identification using Pfam models (Figure 2). The conservation of the two α2-α3 and α4-α5 histidine residues is key, as these conserved histidine imidazoyl side chains bind the heme cofactor in authentic cytochrome b5 proteins.

**Figure 3.**
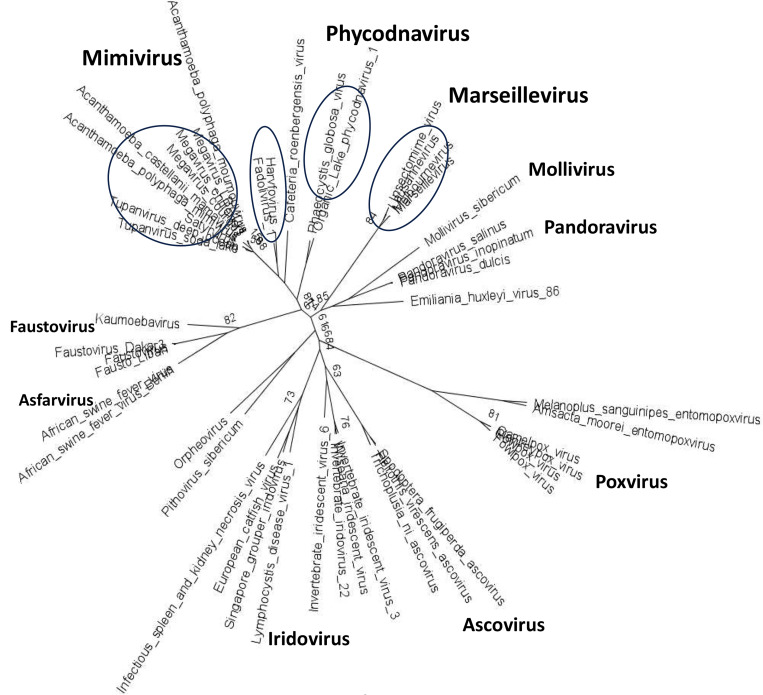
Megavirales phylogeny constructed from the RNAPol2 gene. The maximum likelihood phylogenetic tree shows the relationship of key members of the Megavirales viruses. The clades that contain encoded cytochrome b5 genes are circled. Note that Otv2 does not appear to have a RNAPol2 gene but is a phycodnavirus. Only bootstrap support values below 90% are shown on the branches.

A partial phylogeny of the *Megavirales* with the branches containing cytochrome b5 genes highlighted in Figure 3 and details of the viral cytochromes b5 are presented in Table 1. Thus far, our searches revealed cytochrome b5 genes to be present in specific branches of the *Megavirales*, notably in the *Mimiviridae* (Groups A, B and C) and in Tupanviruses. Cytochrome b5 genes were also detected in giant virus genomes assembled during the analysis of metagenomic data from terrestrial soils (Satyrvirus, Harvfovirus, Hyperionvirus and Terrestrivirus) and from marine sediments (Marseillevirus and Pithovirus) ^30^. In addition, cytochrome b5 genes were retrieved from the genomes of NCLDV that infect the green algae *Ostreococcus lucimarinus and O. tauri* (the Phycodnavirus OtV-2 mentioned above) ^31^. Among the varied proteins with cytochrome b5 domains, the virus cytochrome b5s cluster with authentic cytochrome b5s, lactate dehydrogenase, and fatty acid dehydrogenase proteins (Supplemental Figure).

### 3.2 Mimiviridae cytochromes b5 genes

Searching the first giant virus genome sequence ^2^, Mimivirus APMV (Figure 1A), revealed the presence of a gene (*MIMI_L628*) coding for a 124 amino acid protein containing the canonical eukaryotic cytochrome b5 heme domain (located between residues 49–124). Following this initial observation, we discovered putative cytochrome b5 proteins encoded in giant viruses in all three *Mimiviridae* sublineages: groups A, B and C (Table 1). Analysis of the viral cytochrome b5 sequences revealed the putative proteins to be either soluble in nature, existing as a single hydrophilic domain protein, or as presumed membrane bound proteins possessing a single N-terminal transmembrane anchor. This was true both within and between *Mimiviridae* sublineages. For example, in group A, Mimivirus cytochrome b5 (accession number Q5UR80) consists of a hydrophilic heme-bound globular domain with a predicted 16–18 amino acid N-terminal transmembrane anchor, while the Hirudovirus cytochrome b5 gene encodes a smaller b5 domain without an encoded recognizable membrane anchor (Table 1; Figure 3). Similar examples of membrane bound and soluble cytochromes b5 are observed in sublineage groups B and C (Table 1; Figure 3). The occurrence of an N-terminal membrane anchor is unique to viral cytochrome b5 proteins alone; all eukaryotic cytochrome b5 proteins examined to date have a C-terminal membrane anchor, *e.g.* human and viral amoeba host *A. castellanii* cytochromes b5 (Figure 2).

There is a strikingly high degree of sequence identity (98–100%) between inferred cytochrome b5 protein homologues in Group A Mimiviruses from different parts of the world. Similarly, there is >95 % sequence identity among the group B cytochrome b5 proteins, and among the group C cytochrome b5 proteins, from different parts of the world. Between groups there is lower conserved sequence identity, with group A cytochrome b5s having <60% identity with group B or group C cytochrome b5s. Group B and C cytochromes b5 share a somewhat higher % identity (~75%). All of the *Mimiviridae* cytochrome b5 genes are AT-rich (69–75%), like the *Mimiviridae* genomes overall, and nucleotide sequences also are highly similar within subgroups.

**Figure 4.**
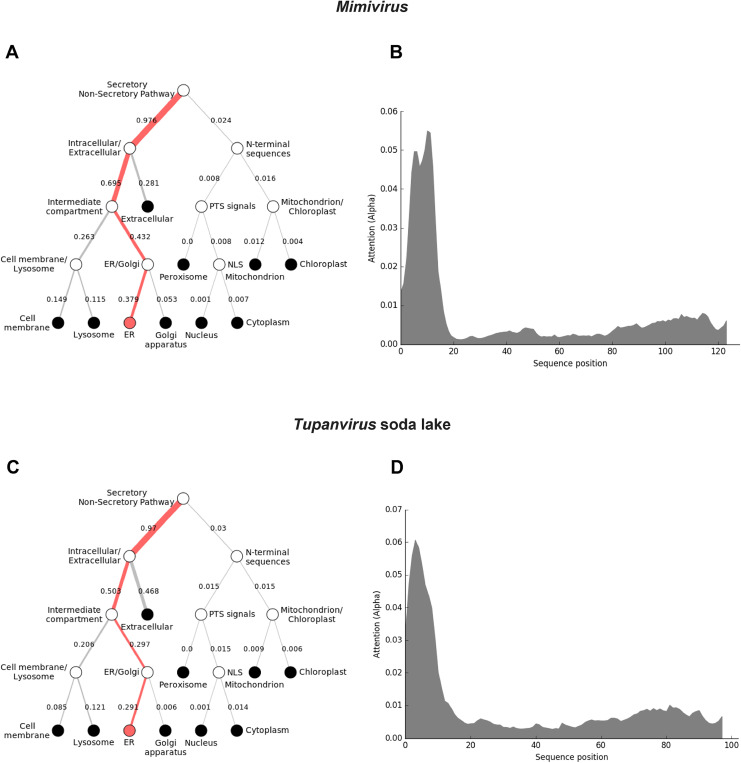
Viral cytochrome b5 subcellular localization predictions. The DeepLoc prediction server indicates that the full-length cytochrome b5 proteins of **(A)** Mimivirus and **(C)** Tupanvirus are highly likely to be localized to the endoplasmic reticulum (ER), with their signal peptides located in their N-terminal sequences **(B, D)**.

To further understand how the *Mimiviridae* cytochrome b5 proteins utilize their N-terminal transmembrane anchor to interact and colocalize with cellular organelles, we performed a protein colocalization prediction. In group A, Mimivirus cytochrome b5 (Q5UR80) is predicted with high likelihood to colocalize with the ER through its N-terminal sequence (Figure 4). However, this was not the case for all the *Mimiviridae* of group A. Some of cytochrome b5s such as in Hirudovirus, Oyster virus, and Samba virus, were predicted to be in the cytoplasm but without association with any specific cellular organelle (Supplemental Table 1). This discrepancy could arise from the absence of a discernible membrane anchor in these proteins. Similar observations are noted within groups B and C (Supplemental Table 1). This finding suggests that viral cytochrome b5 proteins strategically utilize their N-terminal membrane anchor to associate with the ER, potentially implicating these proteins in critical ER-related processes such as lipid and protein synthesis, which are vital for viral replication and assembly.

### 3.3 Tupanvirus cytochromes b5 genes.

Tupanviruses (Figure 1B), related to *Mimiviridae*, were discovered in a soda lake in Brazil and in the deep ocean (3,000-m depth) and are the first viruses reported to possess genes for amino-acyl tRNA synthetases for all 20 standard amino acids ^32^. Tupanvirus genomes contain multiple cytochrome b5 genes. Both the soda lake and the deep ocean Tupanviruses possess genes predicted to encode cytochrome b5 proteins, each containing a N-terminal transmembrane anchor (Supplemental Table 1; Figure 4). While the Tupanvirus cytochromes b5 share 78% percent amino acid identity, they share between 55–60% sequence identity with *Mimiviridae* groups A and B cytochrome b5 proteins but only 45% identity with group C b5 proteins. Subcellular colocalization predictions for both Tupanviruses’ cytochrome b5 proteins showed a strong likelihood of association with the ER, possibly due to an ER-targeted sequence within their N-terminal region (Figure 4). This observation suggests that as with Mimivirus, the Tupanvirus cytochrome b5 proteins are likely to colocalize with the ER, suggesting its involvement in modulating ER function and potentially exerting influence over various metabolic pathways to enhance viral fitness.

Subcellular localization predictions for the cytochrome b5 proteins of *O. lucimarinus* virus, Marseillevirus, and Satyrvirus revealed a different pattern. These proteins did not show significant colocalization with the ER. Instead, our prediction results indicated their localization in the cytoplasm, with a weak likelihood of association with specific organelles (Supplemental Table 1). This discrepancy suggests that the subcellular localization of cytochrome b5 proteins in infected hosts vary among different viruses. One possible explanation for this difference could be the absence of a membrane anchor on these proteins, as indicated by our alignment result (Figure 2). The lack of a membrane anchor could be expected to prevent their association with the ER and other organelles, leading to their predominant localization in the cytoplasm. These findings underscore the complexity of this protein localization and the diverse strategies employed by viruses to interact with cellular machinery.

### 3.4 Gene synteny, transcriptome analysis and cytochrome b5 spectral analysis in Megaviridae.

**Figure 5.**
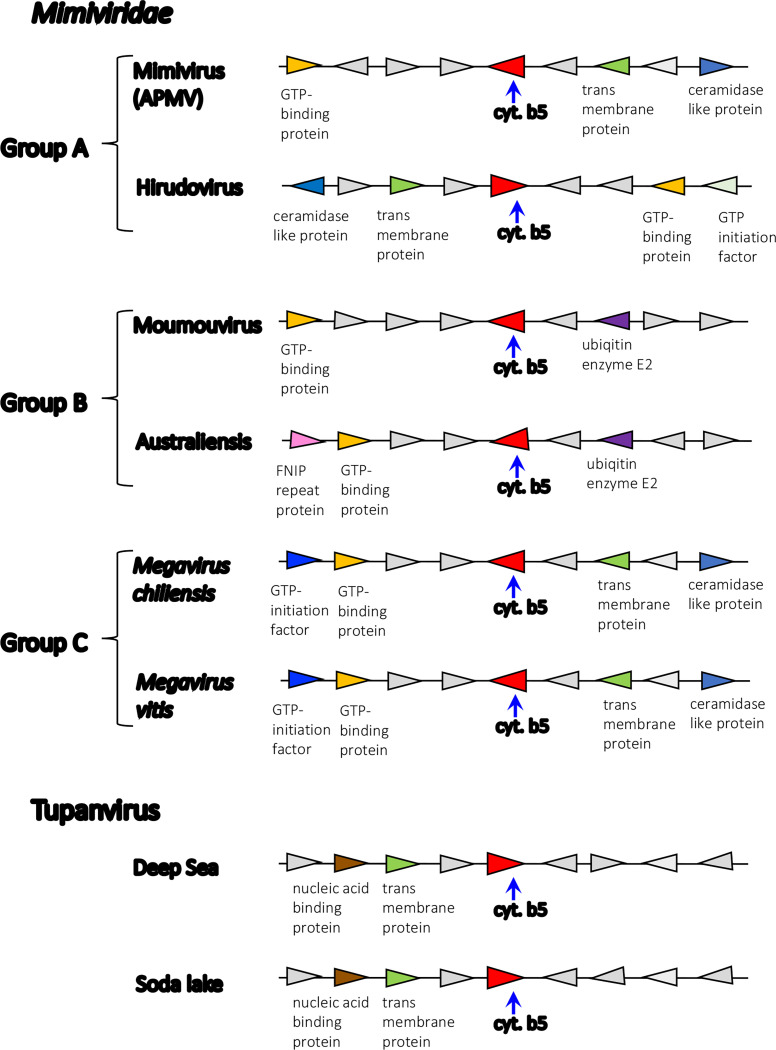
Synteny analysis of cytochrome b5 loci in *Mimiviridae* and Tupanviruses. Comparative examples of the loci and surrounding genes for membrane bound and soluble cytochrome b5s in *Mimiviridae* Group A, B and C viruses, and the membrane bound cytochrome b5s in the soda lake and deep sea Tupanviruses.

**Figure 6.**
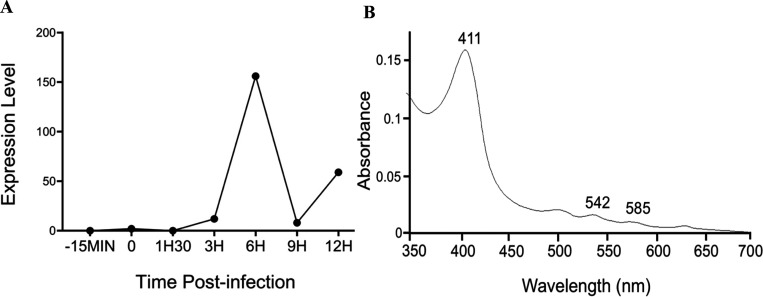
Mimivirus cytochrome b5 transcriptomic profile and protein spectrum characterization. **A.** Cytochrome b5 expression levels during the Mimivirus replication cycle, presented with normalized read counts. **B.** Oxidized absorbance spectrum of purified recombinant Mimivirus L628 cytochrome b5.

*Mimiviridae* groups A, B and C exhibit high degrees of shared gene order (synteny) extending over 8 genes around the cytochrome b5 gene (Figure 5). Transcriptomic analysis also revealed that, of the 90% of all Mimivirus genes transcribed during infection, the Mimivirus gene L628 (which putatively encodes cytochrome b5) is maximally transcribed at 6h (Figure 6A) ^33^. This expression pattern falls into the ‘late’ class of expression upon Mimivirus infection, as do other genes encoding oxidoreductase enzymes, or structural components of the Mimivirus particles ^33^. Heterologous expression of histidine-tagged Mimivirus cytochrome b5 (MIMI_L628) in *E. coli* and purification using nickel affinity chromatography yielded a distinctive hemoprotein. Cell fractionation revealed MIMI L628 to be a membrane bound protein being located in the membrane fraction following ultracentrifugation at 100 000 × *g* and solubilised by the use of detergents (1% (v/v) Tween 20 and 1.5% (w/v) sodium cholate). The absorbance spectra of oxidized recombinant MIMI L628 protein (Figure 6B) shows maxima at 412, 528, and 567 nm. These spectral characteristics are typical of purified cytochrome b5s from eukaryotic organisms described in the literature (18).

### 3.5 Analysis of cytochrome b5 secondary structure and overall topology

**Figure 7.**
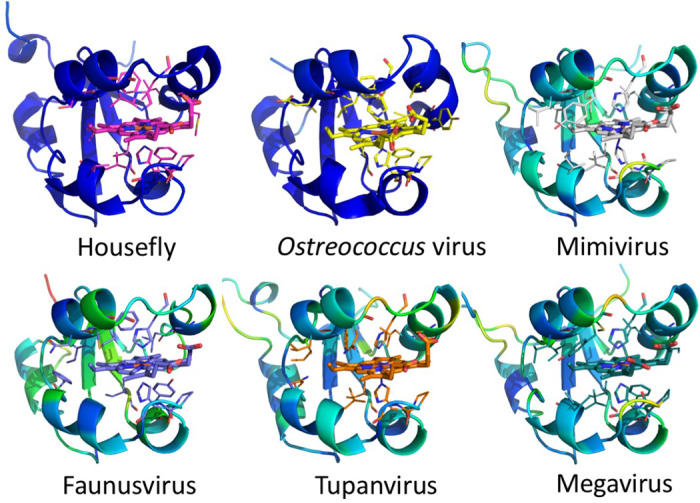
Structures of cytochrome b5 compared with homology models of viral cytochrome b5. The homology model helices are colored by b-factor (blue = highest confidence, red = least confidence).

**Figure 8.**
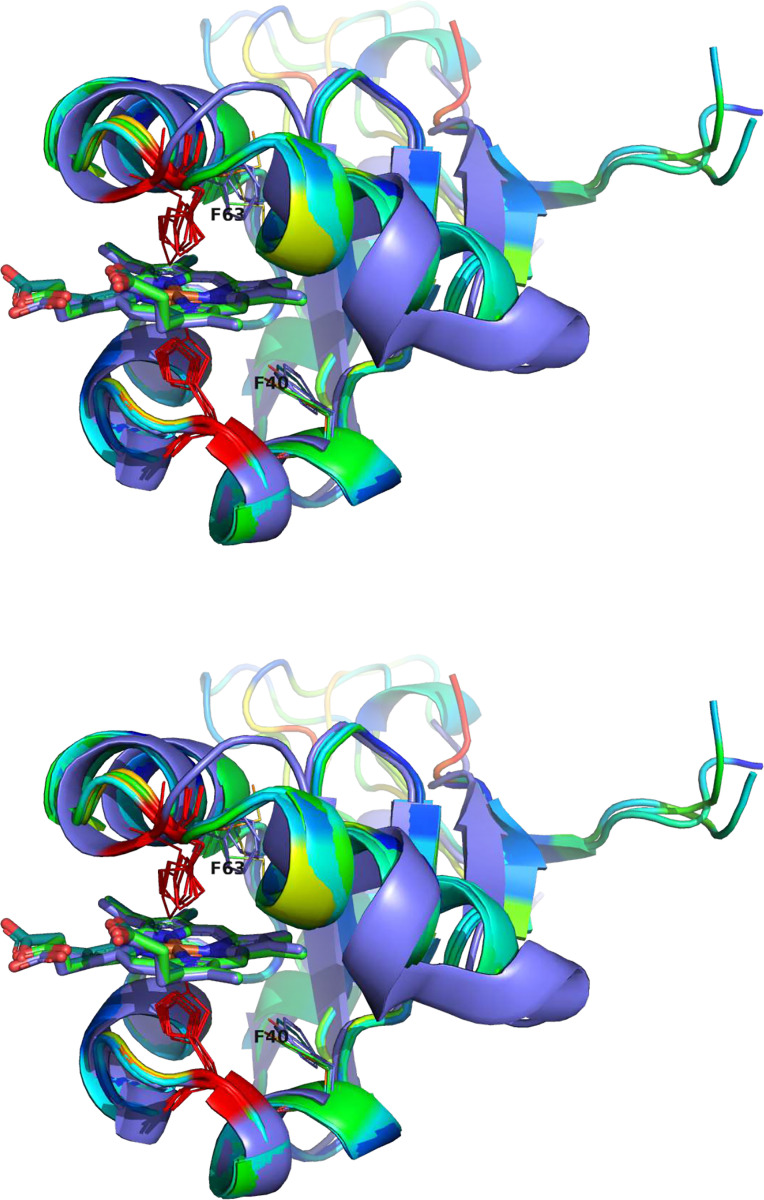
Overlaid viral cytochrome b5 structures showing the positioning of the conserved histidine residues (red) binding the heme. Note that there is some variability in the heme-adjacent residues. Human, amoeba, and virus conserved F40 (alignment position 91) is a tyrosine in Faunusvirus, while F63 (alignment position 114) is a conserved methionine in Mimivirus, Megavirus, Tupanvirus, and Moumouvirus, but a phenylalanine in soil Faunusvirus, Hyperion virus, Terrestrivirus and metazoan cytochrome b5s.

All eukaryotic ER-bound microsomal cytochrome b5 proteins examined to-date are anchored to the ER lipid bilayer by a C-terminal transmembrane anchor. Mitochondrial cytochrome b5 proteins are also anchored in the membrane by a C-terminal transmembrane anchor. In the case of human and *A. castellanii* microsomal cytochrome b5s, each possesses a single 16–18 amino acid C-terminal region that anchors the hydrophilic N-terminal domain to the ER membrane (Figure 2). In contrast, analysis of the viral cytochrome b5 sequences reveals the putative proteins to be either soluble, existing as a single hydrophilic domain protein, or membrane bound apparently through a putative N-terminal transmembrane anchor sequence rather than a C-terminal anchor. Protein modelling demonstrates the high degree of tertiary structural homology between the crystalized house fly and *Ostreococcus* cytochrome b5 and the modelled Mimivirus, Megavirus, Tupanvirus, and Faunusvirus cytochrome b5s (Figure 7). Notably, the heme-binding histidines are spatially conserved (Figure 8), but there is some variability in the heme-adjacent residues: conserved *Ostreococcus* Phe44 is a tyrosine in Faunusvirus; and Phe67 is a conserved methionine in Mimivirus, Megavirus, Tupanvirus, and Moumouvirus, but a phenylalanine in soil Faunusvirus, Hyperionvirus, and Terrestrivirus cytochrome b5s.

## 4. Discussion and Conclusions

Our search of more than ten thousand completely sequenced and annotated viral genomes revealed cytochrome b5 genes in several virus taxa, including the AT-rich genomes in the *Mimiviridae,* Tupanvirus, and *Marseilleviridae,* members of the *Megavirales*, large double stranded DNA viruses. Additionally, we found cytochrome b5 genes in viruses infecting *O. lucimarinus*, similar to the *virus infecting O. tauri* (Table 1). The *Ostreococcus* viruses are also members of the NCLDV virus family, albeit much smaller than the giant Mimiviruses, and lacking RNAPol2 genes. We assigned genes as encoding cytochrome b5 based on the presence of the conserved heme-binding consensus motif found in all described cytochrome b5s ^17^. Although part of the same virus family, the *Mimiviridae* genes are not homologous with the *Ostreococcus* virus genes and appear to have been acquired separately, potentially an example of convergent evolution. While size of genome is a clear and defining feature for giant viruses, it is interesting to note that OtV2 infects one of the world’s smallest free-living eukaryotes, suggesting an important metabolic role(s) for cytochrome b5 in overcoming the extremes of scale in both the virus and host.

The earlier expression of a recombinant cytochrome b5 from the OtV2 virus, and analysis of activity, showed that at least this virus cytochrome b5 gene encodes a functional protein ^20^. Whether cytochrome b5 genes in the other *Ostreococcus* phage and the giant viruses also encode functional proteins is not known yet, although structural and transcriptional features suggest that the viral cytochrome b5 proteins are almost certainly functional. Larger questions regarding functional role(s) these enzymes play in the viruses, or hosts, and the origin of the virus cytochrome b5 genes are as yet unanswered.

Increasingly, a number of metabolic enzymes encoded by genes in the giant viruses, the NCLDV, have been found to be functional *in vitro* and to be expressed *in vivo* upon infection of a host (10,11). These include genes encoding enzymes involved in glycolysis, gluconeogenesis, tricarboxylic acid cycle, photosynthesis, and β-oxidation. One of the important functions of cytochrome b5 is providing reducing equivalents during the catalytic cycle of cytochrome P450 proteins. This could be considered as a possible function in the *Mimiviridae* and the Tupanviruses, where there are multiple P450 genes, and those genes are also expressed in the infected host, shown at least for Mimivirus ^11^. However, the *Marseilleviridae*, which do have cytochrome b5 genes, do not appear to have genes that encode any cytochrome P450. Moreover, our search revealed cytochrome b5 genes only in the AT-rich *Mimiviridae*, *Marseilleviridae* and the Tupanviruses but not in the other branches of the *Megavirales* including in the GC-rich *Pandoraviridae*, Mollivirus, Kaumoebavirus or the Orpheovirus, all of which contain cytochrome P450 genes. This would suggest that if cytochrome b5 does support virus P450 function, that so far it would seem to be restricted to AT-rich virus clades

In addition to its role in the mono-oxygenation by cytochrome P450, cytochrome b5 plays pivotal roles in cellular metabolism, particularly in fatty acid elongation, desaturation, and cholesterol biosynthesis ^34^. These metabolic pathways are essential for maintaining cellular homeostasis and protecting against metabolic disorders ^35^. In the context of viral infection, the presence of cytochrome b5 in giant viruses like Mimiviruses and Tupaniviruses suggests its potential involvement in similar metabolic processes. Our *in silico* predictions of subcellular colocalization indicate cytochrome b5’s likely localization within the endoplasmic reticulum (ER), consistent with its predicted transmembrane domain, and suggests that cytochrome b5 expression in the ER may be related to the ER involvement in lipid metabolism. The ER serves as a hub for lipid metabolism, housing enzymes crucial for triglyceride and cholesterol biosynthesis as well as ergosterol biosynthesis in amoebae ^36^. During the replication cycles of viruses like Mimiviruses, there is a notable close association between membranes derived from the endoplasmic reticulum (ER) and sites where viral assembly occurs ^37^. This suggests that the ER is effectively “hijacked” for lipid synthesis, a process crucial for the formation of virus particle envelopes as shown in some DNA viruses ^38^. Importantly, no viral cytochrome b5 reductase has yet been discovered indicating that the viral cytochrome b5s described herein function in tandem with host cytochrome 5 reductase. Viral cytochrome b5 may serve a function within the ER, participating in the desaturation and elongation of fatty acids. Such a role would ensure the availability of an adequate lipid supply, which is essential for the overall fitness and successful replication of the virus. Moreover, in this case, we speculate that cytochrome b5, by transferring electrons to host enzymes, may facilitate the synthesis of polyunsaturated fatty acids by transferring electrons to host and virally-encoded enzymes involved in lipid metabolism.

Another potential role of cytochrome b5 in giant viruses is as an antioxidant. Viral replication can stress the host cell, leading to reactive oxygen species (ROS) accumulation ^39^ Studies have shown that overexpression of cytochrome b5 in mammalian cells reduces oxidative stress ^40^ implying that cytochrome b5 in viruses might similarly safeguard the viral replication apparatus from ROS-induced damage. Nevertheless, the roles of cytochrome b5 in giant viruses likely span beyond a single function. Given its multifaceted nature, comprehensive molecular and biochemical studies are needed to decode specific contributions of cytochrome b5 within giant viruses, underscoring the need for further investigative work to clarify its exact function and role.

In a previous study, we found it impossible to conclude that the P450 genes in the *Mimiviridae* were acquired from any known host ^21^. There were, however, some indications in other viruses that P450s were acquired from a known host eukaryote. Thus, Hokovirus CYP5724A1 and *Ranid herpesvirus 3* CYP5723A1 share identities of approximately 30% sequence identity to lipid and steroid metabolizing P450s present in their hosts, and the P450 102L1 we reported in the mycophage *Mycobacterium* phage Adler was acquired from the *Mycobacterium abscessus* host ^21^. A similar picture appears with the cytochrome b5 genes. The cytochrome b5 genes in the NCLDV do not appear to have been derived from any known contemporary hosts, in contrast to the cytochromes b5 found in the *Ostreococcus* phages, which do bear significant similarities to the cytochromes b5 in their algal hosts. It would be informative to determine whether there is similarity to cytochrome b5 in any hosts other than amoebae, should such hosts be identified. Barring that, the NCLDV cytochrome b5 genes at the present time do not appear to have originated through recent gene transfer, but rather, as is the case for many Megavirales genes, may have originated in transfer from ancient, no longer extant, hosts.

The major distinction in the presence of cytochrome P450 genes and cytochrome b5 genes among the giant viruses either indicates that these genes were acquired separately, or that if they were acquired together, that the cytochrome b5s were lost from the GC-rich clades. Alternatively, if the cytochrome b5 genes are *de novo* creations of the giant viruses, then that process would have been restricted, or the genes may have been lost along the way from the GC-rich clades. For both cytochrome b5 and cytochrome P450 there is an absolute requirement for heme cofactor, intimating that both viral gene families were transferred from a cellular host as no heme biosynthetic enzyme machinery has been found to be encoded in a giant virus genome to-date. Alternatively, the virus may originally have possessed heme machinery but then lost it as it became dependent on the host pathway for heme supply. In either case, the presence of genes for cytochromes b5 in the giant viruses adds to the growing number of genes in the giant viruses that are not expected in any virus, informing the discussion of the origin of the giant viruses and the genes they contain. In particular, this study raises further a question about the origin of genes for heme proteins and their role in the giant viruses.

## Supplementary Material

Supplement 1

## Figures and Tables

**TABLE 1.** Virus Cytochrome B5 Features

Taxon/Species	Virus origin	Total ORFs	Protein length	Trans-membrane region	Accession number
***Mimiviridae* A**					
Mimivirus	U.K.	1262	124	5–22	Q5UR80
Mamavirus	France	988	118	6–22	AEQ60829
Oyster virus	Brazil	969	89	-	AKI79407
Kroon virus	Brazil	930	86	-	AKI80364
Hirudovirus	Tunisia	930	95	-	AHA45216
Niemeyer virus	Brazil	1003	124	6–23	ALR84216
Bombay virus	India	898	124	6–23	AMZ03071
Shirakomae virus	Japan	986	95	-	BAV62730
Kasaii virus	Japan	988	95	-	BAV61744
Samba virus	Brazil	971	95	-	AHJ40257
***Mimiviridae* B**					
Moumouvirus	France	894	111	12–33	AGC02191
Moumouvirus monve	France	1150	111	12–33	AEX62481
Saudi moumouvirus	S-Arab	953	111	12–33	AQN68565
Moumouvirus goulette	France	970	112	12–33	AGF85059
Moumouv. australiensis	Australia	903	74	-	AVL95051
Moumouvirus maliensis	Mali	845	112	12–33	QGR54182
Borely moumouvirus	Brazil	934	112	12–33	QID06377
***Mimiviridae* C**					
Megavirus chiliensis	Chile	1120	103	6–22	AEQ33147
Mimivirus lba	France	1176	77	-	AGD92703
Megavirus courdo11	France	1337	77	-	AFX92855
Powai Lake Megavirus	India	996	103	6–22	ANB50850
Bandra Megavirus	India	1055	103	6–22	AUV58705
Megavirus vitis	France	1027	77	-	AVL94070
Mimivirus sp SH	China	947	103	6–22	AZL89108
Tupanvirus (Deep Ocean)	Brazil	1276	98	1–18	QKU33443
Tupanvirus (Soda Lake)	Brazil	1359	98	1–21	QKU34676
Satyrvirus sp. (metagenome)	USA	906	76	-	AYV84960
Marseillevirus LCMAC102 (metagenome)	Marine sediment	456	78	-	QBK86278
*O. lucimarinus Virus* Olv1	France	250	91	-	ADQ91589
Olv2	USA	269	92	-	YP_009172721
Olv3	Chile	248	92	-	AFK66042
Olv4	Canada	299	91	-	AET84663
Olv5	France	251	91	-	AGH31115
Olv6	France	248	91	-	AFK65792
Olv7	USA	243	91	-	ALI95841
*O. tauri* virus OtV2	Ocean	237	91	-	CB170200
*Homo sapiens*	-	~20,000	134	113–129	NP_683725
*Acanthamoeba castellanii* Neff		15,455	141	119–137	ELR24118
