# Supplementary material for: Cytochromes b5 Occurrence in Viruses Belonging to the Order Megavirales
Source: Res Sq. 2024 Oct 25:rs.3.rs-5246363. Preprint. [Version 1] doi: 10.21203/rs.3.rs-5246363/v1 (PMC11537341; doi:10.21203/rs.3.rs-5246363/v1)
Supplement: Supplement 1 [file NIHPPRS5246363V1-supplement-1.pdf]

Supplementary Files

This is a list of supplementary files associated with this preprint. Click to download.

- Lambb5SupplementalTable1.pdf
- Lambb5supplementalFigure1.pdf
